# Mapping the Landscape of Over‐Scanning in CT Imaging: A Scoping Review

**DOI:** 10.1002/jmrs.70104

**Published:** 2026-06-30

**Authors:** Mo'men Bani‐Ahmad, Aoife O Sullivan, Yasser H. Hadi, Laura McLaughlin, Andrew England, Mark McEntee

**Affiliations:** ^1^ Discipline of Medical Imaging and Radiation Therapy University College Cork Cork Ireland; ^2^ Department of Medical Imaging, Faculty of Applied Medical Sciences The Hashemite University Zarqa Jordan; ^3^ Department of Medical Imaging and Intervention King Abdullah Medical City (KAMC) Makkah Saudi Arabia; ^4^ Health Sciences Research Centre UCL University College Odense Denmark; ^5^ Faculty of Health Sciences Syddansk Universitet ‐ University of Southern Denmark Odense Denmark; ^6^ Faculty of Medicine University of Sydney Sydney New South Wales Australia

**Keywords:** computed tomography, over‐scanning, protocol standardisation, radiation dose, scan range

## Abstract

**Introduction:**

Over‐scanning in CT refers to extending the scan range beyond intended anatomical boundaries, resulting in unnecessary ionising radiation exposure. Despite its frequent occurrence, it remains poorly defined and inadequately addressed within the radiological community. This scoping review examines the extent of over‐scanning in CT imaging, including its definitions, assessment methods, contributing factors and mitigation strategies.

**Methods:**

A scoping review was conducted in accordance with PRISMA‐ScR guidelines. PubMed, Embase, Ovid and Scopus were searched for studies published between January 2010 and December 2025. Extracted data included definitions, assessment methods, prevalence, dose implications, contributing factors and mitigation strategies.

**Results:**

Thirty‐eight studies covering brain, chest, abdominal and multi‐region CT protocols were included. Over‐scanning was prevalent, averaging 75% (range 13%–100%), with excess coverage from 12 mm to about 90 mm, exceeding recommended anatomical margins. Associated effective dose increases ranged from 0.03 to 3.4 mSv, with organ‐specific doses markedly higher in paediatric imaging and radiosensitive organs (thyroid, lungs, breasts, testes), amplifying long‐term cancer risk. Directional patterns (superior/inferior) varied by protocol, influenced by anatomical complexity, scout‐image limitations and radiographer caution to avoid under‐coverage.

**Conclusion:**

Over‐scanning in CT is a multifactorial issue driven by technical, operator, patient and system‐level factors. Mitigation requires standardised protocols, radiographer training, education on over‐irradiation risks, optimised scout image utilisation and technological support. Key strategies include consistent osseous landmarks, lateral scout views and AI‐based planning tools, which offer promising solutions for automating scan range planning, detecting and reducing over‐scanning through real‐time monitoring and retrospective analysis and enhancing patient radiation safety.

## Graphical abstract

Over‐scanning in CT is highly prevalent and contributes to unnecessary radiation exposure, with notable impact on radiosensitive organs. Standardised protocols and AI‐assisted planning show strong potential to optimise scan range and reduce excess dose.

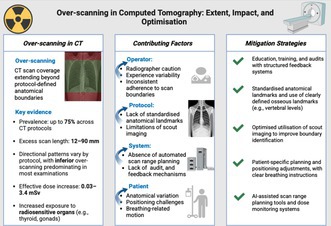

## 1 Introduction

Computed tomography (CT) is a cornerstone of diagnostic imaging, widely employed for disease detection, staging and treatment planning [1]. As its clinical applications expand, concerns about cumulative ionising radiation exposure have intensified, particularly for patients undergoing repeated imaging (e.g., CT for recurrent renal colic, annual low‐dose chest CT for lung cancer screening) or those with heightened radiosensitivity (e.g., paediatric patients) [2, 3]. Epidemiological studies suggest that cumulative doses exceeding 100 mSv may be associated with a measurable increase in cancer risk, with CT imaging potentially contributing to up to 5% of new annual cancer cases if current usage trends persist [4, 5]. Among the modifiable contributors to radiation dose, scan range selection, specifically the extent of *z*‐axis coverage, plays a pivotal role in dose optimisation [6].

In routine clinical practice, the scan range is typically planned manually using scout/localiser images [7]. To ensure complete anatomical coverage, operators often extend the scan range beyond protocol‐defined anatomical boundaries, a precautionary measure termed over‐scanning [8]. This operator‐driven extension is distinct from the equipment‐related phenomena of over‐ranging, which is the additional irradiated length inherently produced at the start and end of helical acquisitions to permit interpolation and reconstruction, and over‐beaming, where the x‐ray beam width exceeds the active detector width and yields unused penumbra dose [9]. Unlike these technical limitations inherent to helical acquisition and beam geometry, over‐scanning is avoidable. It reflects planning decisions and is influenced by anatomical uncertainty, variability in scan‐range prescription and discrepancies arising from respiratory motion during breath‐hold instructions, such as in the chest or upper abdomen [10]. Although precautionary extension aims to prevent under‐coverage, it frequently exposes adjacent tissues to unnecessary ionising radiation [11]. To balance diagnostic adequacy with radiation protection, acceptable tolerance in the literature is specified either as fixed margins (typically 10–20 mm per direction, cranially and caudally) or as a percentage of the total scan length, most commonly 10%, in alignment with the As Low As Reasonably Achievable (ALARA) principle [10]. However, adherence to these margins remains inconsistent across institutions and radiographer practices, and the literature varies in how scan coverage adequacy and associated dose implications are defined and assessed [12, 13].

Over‐scanning is common across CT protocols and anatomical regions, yet its prevalence, contributing factors and implications for radiation dose remain inconsistently documented [14, 15]. These gaps are particularly concerning in paediatric imaging, where radiosensitive organs are more vulnerable and in multi‐region scans where cumulative exposure can be substantial. A comprehensive synthesis of current evidence is therefore needed to better understand the extent of over‐scanning, radiation dose and suggested strategies for mitigation.

This scoping review aims to systematically map the landscape of over‐scanning in CT imaging, including its definitions, assessment methods, prevalence and dose implications. By synthesising available data, the review seeks to clarify the extent of over‐scanning, examine contributing factors and collate mitigation strategies to support safer and more standardised scan planning practices.

## 2 Methods

This scoping review was conducted in accordance with the PRISMA‐ScR methodological framework to ensure transparency and reproducibility [16]. To support methodological rigour, the completed PRISMA‐ScR checklist is provided in Table S1.

### 2.1 Search Strategy

A systematic search was conducted across four electronic databases: PubMed, Ovid, Embase and Scopus, to identify relevant studies published between January 2010 and December 2025. The search strategy used free‐text terms related to CT, scan range and radiation dose. Boolean operators (AND, OR) were applied to refine the results. Full search strings and the number of retrieved records per database are presented in Table S2. In addition to database searching, citation screening of the reference lists of included studies was performed to identify additional relevant articles.

### 2.2 Study Eligibility Criteria

Eligibility criteria were defined to align with the objectives of this scoping review, which focused on over‐scanning in CT imaging. Studies were eligible if they examined CT imaging in any anatomical region and reported empirical data related to scan range, scan coverage, or over‐scanning (whether assessed quantitatively or qualitatively). Both manual and automated assessment methods were included. Studies were excluded if they were not CT‐based, focused solely on other technical parameters (e.g., tube current/potential), or were review articles, conference abstracts, editorials, or non‐English publications.

### 2.3 Study Selection and Data Extraction

All retrieved citations were imported into Covidence (Covidence, Melbourne, Australia) for screening and data management. Duplicate records were automatically removed. Two reviewers (M.B.A. and A.O.S.) independently screened titles and abstracts to identify potentially eligible studies based on the predefined inclusion and exclusion criteria. Articles were excluded during screening if they did not clearly address scan coverage or over‐scanning in CT imaging. Full‐text articles were then assessed against the inclusion criteria. Disagreements were resolved through discussion between the two reviewers, with arbitration by a third reviewer (Y.H.) when consensus could not be reached. Data extraction was performed manually using structured forms. Extracted variables included, where applicable, study characteristics (author, year, design, setting, sample size), anatomical region, definition and assessment of over‐scanning, extent and prevalence, radiation dose metrics (e.g., effective dose), contributing factors and mitigation strategies.

### 2.4 Data Synthesis and Visualisation

Over‐scanning extent, prevalence and ionising radiation dose were summarised descriptively for each CT protocol using ranges and frequencies reported in the included studies. Over‐scanning extent was expressed in millimetres (mm), prevalence as the percentage of over‐scanned cases and radiation dose in millisieverts (mSv). Dose quantities were extracted exactly as reported in the source studies and labelled consistently. Organ absorbed dose was recorded in milligray (mGy) when studies modelled or reported absorbed dose to specific organs and organ equivalent dose in mSv when values were produced by dosimetry software implementing Monte Carlo modelling with the International Commission on Radiological Protection (ICRP) tissue weighting [17]. Effective dose (mSv) was documented as published by each study and was derived either from modelling organ/tissue doses with ICRP 103 tissue weighting or by conversion from dose–length product (DLP, mGy·cm) using region‐specific k factors [18]. Entries without reported effective dose were marked as not reported (NR). Over‐scanning contributing factors reported in the included studies were coded under categories developed from the data. If a study reported multiple categories or factors, all were counted. Percentages were calculated based on the frequency of mentions within each category to illustrate common themes.

## 3 Results

### 3.1 Characteristics of Included Studies

**FIGURE 1:**
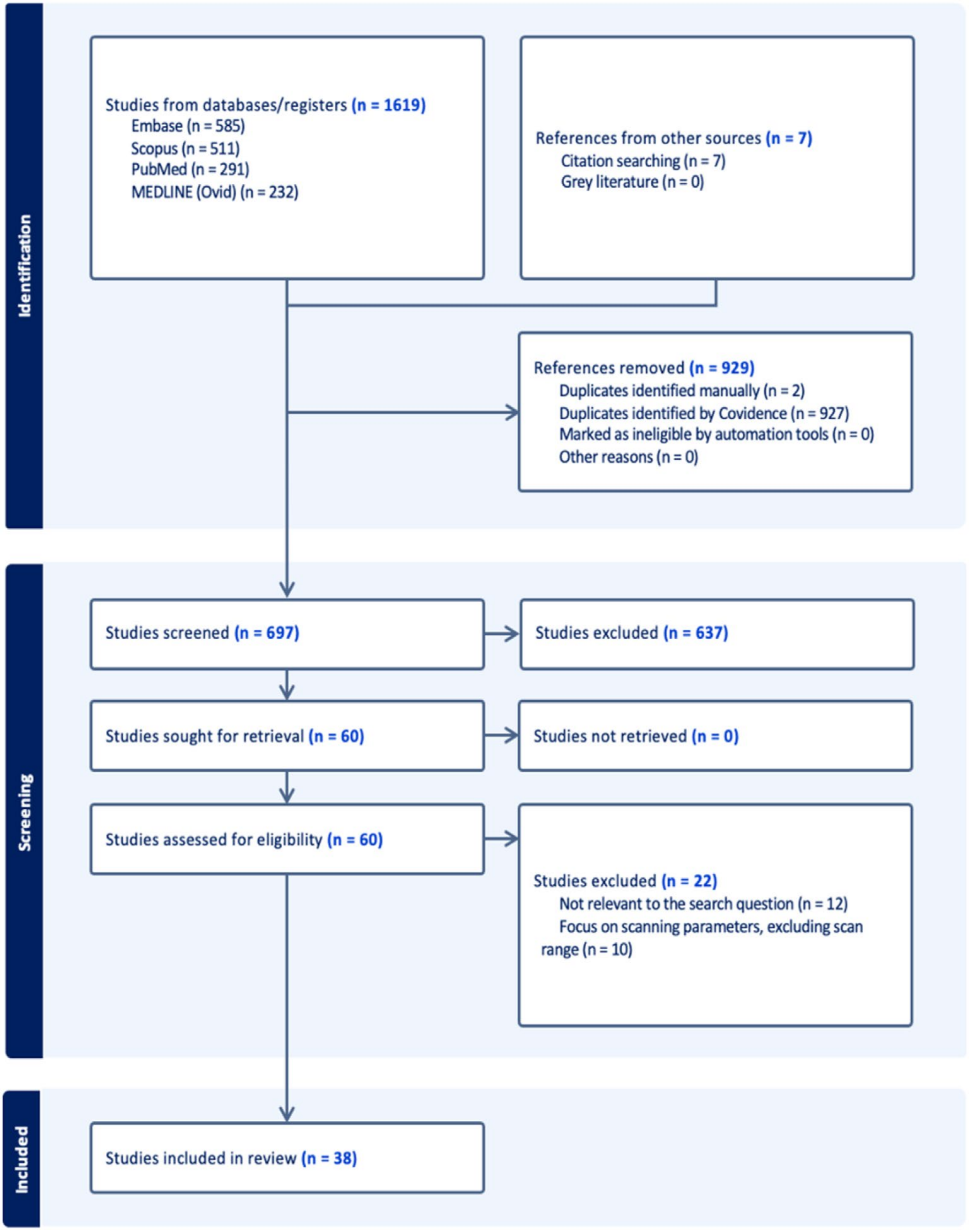
PRISMA flow diagram.

Thirty‐eight studies published between 2010 and 2025 were included (Figure 1), representing a wide geographical distribution across Europe, Asia, the Middle East, North America, South America and Australia. Sample sizes ranged from small single‐centre audits of 54 examinations to large multicentre cohorts exceeding 20,000 scans. Most studies used retrospective observational designs (primarily clinical audits and cohort analyses), reflecting real‐world clinical practice. The included evidence spanned a diverse range of CT protocols, including head [19, 20, 21, 22], neck [23], computed tomography of the kidneys, ureters and bladder (CT‐KUB) [24, 25, 26, 27, 28, 29, 30, 31, 32], chest [33, 34, 35, 36, 37, 38, 39, 40, 41, 42, 43], abdominal, abdominopelvic and combined chest–abdomen–pelvis [44, 45, 46, 47, 48, 49, 50, 51], as well as specialised applications such as coronary CT angiography (CCTA) and CT pulmonary angiography (CTPA) [52, 53, 54, 55, 56]. Paediatric imaging was addressed in several studies, predominantly in head, chest and abdominopelvic examinations, with age groups ranging from neonates to adolescents [19, 20, 22]. Multicentre studies were typically associated with optimisation initiatives, whereas single‐centre studies more often evaluated local practice, protocol adherence or targeted quality‐improvement interventions. The included studies varied substantially in design, clinical indications, populations and protocol types, reflecting the broad range of real‐world scenarios in which CT examinations are performed. Detailed study characteristics are summarised in Table 1.

**TABLE 1:** Characteristics of the included studies.

Author(s) and year	Multi‐/Single centre	Sample size	Setting	Protocol type
Zadeh et al. [19] 2025	Multi‐centre (3)	102	Routine paediatric head CT	Head CT
Woo et al. [20] 2013	Single	54	Routine paediatric brain imaging; and craniosynostosis	Head CT
Botwe et al. [21] 2022	Multi‐centre (25)	1640	Routine diagnostic CT: brain lesion, lung lesion, Pulmonary embolism, Abdominopelvic lesion	Head, chest, pulmonary CT angiography (CTPA), abdominopelvic CT
Ampofo et al. [22] 2025	Multi‐centre (2)	279	Routine paediatric CT scans excluding metastasis, hydrocephalus and trauma	Head, chest, abdominopelvic CT
Badawy et al. [23] 2019	Single‐centre	102	Routine diagnostic	Neck CT
Blom et al. [24] 2025	Single‐centre	247	Renal colic/urolithiasis	Non‐contrast low‐dose CT for kidney, ureter and bladder (CT‐KUB)
Alrosan et al. [25] 2024	Multi‐centre (3)	88	Renal colic/urolithiasis	CT‐KUB
Zafar et al. [26] 2024	Multi‐centre (12)	1265	Renal colic/urolithiasis	CT‐KUB
Owien et al. [27] 2024	Single‐centre	299	Renal colic/urolithiasis	CT‐KUB
Ghoshal et al. [28] 2021	Multi‐centre (4)	88	Renal colic/urolithiasis	CT‐KUB
Kasi et al. [29] 2021	Single‐centre	150	Renal colic/urolithiasis	CT‐KUB
Netke et al. [30] 2020	Single‐centre	150	Renal colic/urolithiasis	CT‐KUB
Uldin et al. [31] 2020	Multi‐centre (3)	194	Renal colic/urolithiasis	CT‐KUB
Leon et al. [32] 2014	Single‐centre	290	Renal colic/urolithiasis	CT‐KUB
Ebrahimian et al. [33] 2021	Multi‐centre (4)	400	Chest CT for suspected or confirmed COVID‐19 and pneumonia	Chest CT
Cohen et al. [34] 2020	Single‐centre	119	Routine diagnostic	Chest CT
Schwartz et al. [35] 2018	Multi‐centre (6)	600	Routine diagnostic	Chest CT
Bang et al. [36] 2013	Single‐centre	210	Routine diagnostic	Low‐dose chest CT
Salimi et al. [37] 2021	Multi‐centre (9)	20,820	Diagnostic COVID‐19 and multiple pathologies.	Chest CT
Huo et al. [38] 2019	Single‐centre	770	Screening	Low‐dose chest CT
Colevray et al. [39] 2019	Single‐centre	1000	Screening	Low‐dose chest CT
Demircioğlu et al. [40] 2021	Single‐centre	1149	Routine diagnostic	Chest CT
Kaviani et al. [41] 2022	Multi‐centre (4)	428	Routine diagnostic	Chest CT
Ruan et al. [42] 2022	Single‐centre	1984	Screening	Low‐dose chest CT
Kim et al. [43] 2022	Multi‐centre (48)	340	Screening	Low‐dose chest CT
Campelo et al. [44] 2020	Multi‐centre (17)	300	Routine diagnostic	Abdominal CT
Luu et al. [45] 2022	Single‐centre	657	Liver cancer	Abdominal & liver CT
Zhang et al. [46] 2015	Single‐centre	264	Routine diagnostic	Abdominopelvic CT
Salerno et al. [47] 2019	Multi‐centre (2)	199	Colonography	Low‐dose CT colonography (CTC)
Golbus et al. [48] 2024	Single‐centre	61	Pulmonary abnormality, malignancy, multisystem autoimmune diseases, immunodeficiency and infectious	Chest, abdominal and abdominopelvic CT
Yar et al. [49] 2021	Single‐centre	1531	Oncologic settings, benign disorders, emergency settings, trauma, postoperative settings and conscious deterioration	Chest CT, abdominopelvic CT, chest‐ abdomin‐pelvis CT, CTPA and CT‐KUB
Zanca et al. [50] 2012	Single‐centre	167	Routine diagnostic	Chest CT, abdominal CT and chest–abdominal CT
Liao et al. [51] 2011	Single‐centre	304	Neoplastic (e.g., cancer staging or follow‐up) Non‐neoplastic diseases (e.g., infection, inflammation, trauma)	Chest, abdomen and/or pelvis CT
Rodrigues et al. [52] 2013	Single‐centre	100	Suspected pulmonary embolism in ED patients	CTPA
Leschka et al. [53] 2010	Single‐centre	125	Coronary artery disease	Coronary CT angiography (CCTA)
Duerden et al. [54] 2022	Single‐centre	95	Suspected coronary artery disease	CCTA
Demircioğlu et al. [55] 2024	Multi‐centre (2)	2038	Suspected coronary artery disease	CCTA
Young et al. [56] 2020	Single‐centre	200	Suspected coronary artery disease	CCTA

Abbreviations: CCTA, coronary CT angiography; CT, computed tomography; CT‐KUB, computed tomography of the kidneys, ureters and bladder; CTC, CT colonography; CTPA, CT pulmonary angiography; ED, emergency department.

### 3.2 Definitions and Assessment Methods

Definitions of over‐scanning varied substantially across the included studies. Most defined over‐scanning as imaging extending beyond protocol‐specified anatomical boundaries, although allowable tolerance ranged from none [19, 20], to allowances of 10–20 mm per direction (cranially and caudally) [49, 52], or proportional thresholds such as 10% of total scan length [25, 27]. Assessment approaches also differed: manual landmark‐based measurements on axial [21, 22, 23] or multiplanar reformatted images were most common [29, 52], while some studies used scout‐view comparisons [24, 35], slice‐based calculations [26, 27], or table‐position data [32]. A smaller number inferred excess coverage from discrepancies between expected and recorded CTDIvol or DLP values [20]. More recent studies applied artificial intelligence (AI) models to predict anatomical boundaries automatically and quantify deviations from optimal coverage [40, 42]. Although authoritative bodies such as the American Association of Physicists in Medicine (AAPM) and the ICRP do not provide specific numerical tolerance margins for acceptable scan‐range extension, both emphasise minimising unnecessary *z*‐axis coverage as part of CT quality control and dose optimisation in line with the ALARA principle [57, 58, 59]. Overall, the heterogeneity in definitions, anatomical landmarks and measurement techniques highlights the lack of a standardised framework for determining excessive *z*‐axis coverage across CT protocols. Definitions of over‐scanning, acceptable tolerance and the methods used for assessment are summarised in Table 2.

**TABLE 2:** Over‐scanning definitions, acceptable tolerance and assessment methods.

Author(s) and year	Protocol type	Definition of over‐scanning & acceptable tolerance	Method of over‐scanning assessment
Zadeh et al. [19] 2025	Head CT	Scan coverage extending caudally beyond the skull base Acceptable tolerance: None	Retrospective analysis of axial CT images: excess scan length measured as the difference between actual and standard coverage
Woo et al. [20] 2013	Brain CT	Scan coverage extending beyond the skull vertex superiorly to below the skull base inferiorly Acceptable tolerance: None	Retrospective analysis of radiation dose values: over‐scanning was quantified by identifying elevated DLP values with normal CTDI_vol_ and confirmed by image review showing the scan range extending beyond the skull base
Botwe et al. [21] 2022	Brain, chest, pulmonary CT angiography (CTPA), abdominopelvic CT	Scanning beyond predefined anatomical boundaries: Brain: skull vertex to foramen magnum (no tolerance) Chest: 10 mm above the apex to 10 mm below the costophrenic angle CTPA: lung apex to costophrenic angle (no tolerance) Abd/Pelvis: 10 mm above the diaphragm to the symphysis pubis	Retrospective analysis of axial CT images: manual measurement of scan length using predefined anatomical landmarks
Ampofo et al. [22] 2025	Brain, chest, abdominopelvic CT	Scanning beyond recommended anatomical boundaries: Head: Skull vertex to 10 mm below the skull base Chest: 10 mm above the lung apices to 20 mm below the costophrenic angle Abdomino‐pelvic: 10 mm above the diaphragm to 10 mm below the pubic symphysis	Retrospective analysis of axial CT images: manual measurement of scan length using predefined anatomical landmarks
Badawy et al. [23] 2019	Neck CT	Scan range above the inferior margin of the sphenoid sinus and below the aortopulmonary window Acceptable tolerance: 20 mm per direction	Retrospective analysis of axial CT images: manual measurement of scan length using predefined anatomical landmarks
Blom et al. [24] 2025	Non‐contrast low‐dose CT for kidney, ureter and bladder (CT‐KUB)	Over‐scanning is defined as slices beyond the T10 endplate and inferior margin of the symphysis pubis Acceptable tolerance: None	Retrospective analysis of scout CT images: manual measurement of scan length using predefined anatomical landmarks
Alrosan et al. [25] 2024	CT‐KUB	Scan length above the highest kidney and below the pubic symphysis Acceptable tolerance: ≤ 10% of the total scan length above the highest kidney	Retrospective analysis of axial CT images: manual measurement of scan length using predefined anatomical landmarks
Zafar et al. [26] 2024	CT‐KUB	Over‐scanning was defined as slices above the upper pole of the highest kidney Acceptable tolerance: None	Retrospective analysis, slice‐based calculation: manual measurement of scan length by counting slices above the highest kidney and below the pubic symphysis
Owien et al. [27] 2024	CT‐KUB	Scan extent beyond the highest kidney and below the pubic symphysis Acceptable tolerance: 10% of the required scan range	Retrospective analysis slice‐based calculation: manual measurement of scan length by counting slices above the highest kidney and below the pubic symphysis, to the total number of slices
Ghoshal et al. [28] 2021	CT‐KUB	Scan length above the highest kidney and below the pubic symphysis Acceptable tolerance: ≤ 10% of the total scan length above the highest kidney	Retrospective analysis of axial CT images: manual measurement of scan length using predefined anatomical landmarks
Kasi et al. [29] 2021	CT‐KUB	Scan extent beyond the superior border of the kidneys and the inferior border of the pubic symphysis Acceptable tolerance: 20 mm per direction	Retrospective analysis of multiplanar reformatted CT images: manual measurement of scan length using predefined anatomical landmarks
Netke et al. [30] 2020	CT‐KUB	Excess scan length above the upper pole of the highest kidney and below the pubic symphysis Acceptable tolerance: ≤ 10% of the total scan length above the highest kidney	Retrospective analysis slice‐based calculation: manual measurement of scan length as the percentage of excess slices above the highest kidney and below the pubic symphysis relative to the total number of slices
Uldin et al. [31] 2020	CT‐KUB	Scan extends beyond the upper limit of the kidneys Acceptable tolerance: ≤ 10% of the total scan length above the highest kidney	Retrospective analysis of axial CT images: manual measurement of scan length as the percentage of coverage extending superior to the kidney margins relative to the total scan length, using anatomical reference to the diaphragm
Leon et al. [32] 2014	CT‐KUB	Over‐scanning was defined as the presence of more than one additional slice above the superior margin of the kidneys or below the inferior margin of the pubic symphysis Acceptable tolerance: 1 slice beyond the defined anatomical boundaries	Retrospective analysis length‐based calculation: manual measurement of over‐scanning as the extension beyond predefined cephalad and caudal scan boundaries using table position data
Ebrahimian et al. [33] 2021	Chest CT	Scan length above the C7/T1 level and below the T12/L1 level Acceptable tolerance: None	Retrospective analysis of axial CT images: manual measurement of scan length using predefined anatomical landmarks
Cohen et al. [34] 2020	Chest CT	Any scan extending above the lung apices or below the lung bases Acceptable Tolerance: ≤ 20 mm per direction	Retrospective analysis slice‐based calculation: manual measurement of scan length by counting slices above and below the predefined anatomical landmarks and multiplying by slice thickness
Schwartz et al. [35] 2018	Chest CT	Any scan range exceeding the upper or lower scan borders of the lungs, based on scout image evaluation Acceptable tolerance: ≤ 20 mm per direction	Retrospective analysis of scout CT images: manual measurement of scan length using predefined anatomical landmarks
Bang et al. [36] 2013	Chest CT	Imaging beyond the lung parenchyma (the upper margin of the first costovertebral joint to the costophrenic angle) Acceptable tolerance: None	Retrospective analysis of axial CT images: redundant imaging quantified by subtracting lung parenchyma slices from total slices and multiplying by slice thickness, comparing planning based on anteroposterior, lateral, or combined topograms
Salimi et al. [37] 2021	Chest CT	Over‐scanning was defined as scan coverage extending beyond the lung apices or costophrenic sinuses Acceptable tolerance: None	Deep learning‐based analysis of 2D scout views: automated prediction of lung boundaries and scan range using convolutional neural networks, comparing technologist‐selected ranges to ground truth derived from 3D segmentation
Huo et al. [38] 2019	Chest CT	Extra scan length beyond the segmented lung region (lung apices to costophrenic sinuses) Acceptable tolerance None	Machine learning‐based analysis of CT localiser images: U‐Net segmentation used to predict lung boundaries and calculate desired scan length; actual axial scan coverage compared to predicted coverage to quantify over‐scanning
Colevray et al. [39] 2019	Chest CT	Scanning beyond lung boundaries (apices to costodiaphragmatic sinus) Acceptable tolerance: 20 mm per direction	Deep learning‐based classification of axial CT slices: cona volutional neural network categorised slices into cervical, thoracic and abdominal regions; over‐scanning quantified as the proportion of slices outside the thoracic region relative to the total scan length
Demircioğlu et al. [40] 2021	Chest CT	A scan range exceeding the true lung borders (lung apices to just below the costophrenic recesses) Acceptable tolerance: 20 mm per direction	Deep learning‐based scan range planning using topograms: conditional generative adversarial network (CGAN) predicts scan start and end positions; automated range compared to radiographers' manual prescription; over‐scanning reduction achieved through shorter AI‐generated scan lengths
Kaviani et al. [41] 2022	Chest CT	Scanning beyond lung boundaries (apices to costodiaphragmatic sinus) Acceptable tolerance: 25 mm per direction	Deep learning‐assisted classification of axial CT slices: physician‐trained DL models used to identify lung apices and bases; classify scans as optimal, under‐scanned, or over‐scanned; over‐scanning defined as > 2.5 cm extension beyond anatomical landmarks
Ruan et al. [42] 2022	Chest CT	Scan coverage beyond the actual lung boundary Acceptable tolerance: 20 mm per direction	Deep learning‐assisted landmark detection on frontal scout images: modified U‐Net with ResNet‐34 encoder identifies pulmonary apices and costophrenic angles; scan range adjusted using 95th percentile of observed distances; over‐scanning assessed by deviation from actual lung boundaries
Kim et al. [43] 2022	Chest CT	Scanning beyond anatomical boundaries from the thyroid cartilage superiorly to the kidneys inferiorly Acceptable tolerance: thresholds of 9 mm (superior) and 3 mm (inferior)	Deep learning‐assisted three‐stage algorithm: automated landmark segmentation using U‐Net, rule‐based logical operations for landmark localisation and over‐scanning detection based on deviation from predefined scan limits (thyroid cartilage to kidney)
Campelo et al. [44] 2020	Abdominal CT	Acquisition of a scout or scan range larger than the anatomical region required for diagnosis Acceptable tolerance: None	Retrospective analysis of scout CT images: excess scan length measured as the difference between actual and standard coverage
Luu et al. [45] 2022	Liver CT	Scan coverage beyond the actual anatomical liver boundaries Acceptable tolerance: 5.9 mm (upper), 15.3 mm (lower) based on Gaussian liver motion modelling	Deep learning‐based scan range planning for multiphase liver CT: 2D CNN liver detector (YOLOv4) combined with liver range search algorithm and Gaussian motion models; scan range automatically generated from scout image with added safety margins; over‐scanning assessed by comparing AI‐generated range to manual radiographer‐defined range
Zhang et al. [46] 2015	Abdominopelvic CT	Scans extended beyond the upper limit above the superior‐most aspect of the left or right hemidiaphragm, or the inferior‐most aspect of the left or right ischial tuberosity Acceptable tolerance: 20 mm per direction	AI‐based classification of abdomen–pelvis CT scans: feedforward artificial neural network (ANN) trained on eight patient and imaging attributes to classify scans as excessive or non‐excessive *Z*‐axis coverage; over‐scanning defined as coverage > 2 cm beyond anatomical landmarks
Salerno et al. [47] 2019	Low‐dose CT colonography (CTC)	Inferior pubic rami as the lower boundary; slices below this are considered over‐scanning Acceptable tolerance: None	Retrospective analysis slice‐based calculation: manual measurement of scan length as the number of CT slices acquired below the standard anatomical boundary
Golbus et al. [48] 2024	Chest, Abdominal and Abdominopelvic CT	Scan range exceeding the optimal anatomical boundaries: Chest CT: the lung apices to the adrenal glands Abdomen CT: the diaphragm through the liver Chest/Abdomen/Pelvis CT: the lung apices through the symphysis pubis Acceptable tolerance: None	AI‐assisted scan range planning using ultra‐low‐dose helical CT scout (3D Landmark Scan) and Anatomic Landmark Detection (ALD): acquisition length automatically set based on detected anatomical landmarks; over‐scanning assessed by comparing acquisition lengths between traditional scout‐based manual planning by ra adiographer and AI‐based planning
Yar et al. [49] 2021	Chest CT, abdominopelvic CT, chest‐ abdomin‐pelvis CT, CTPA and CT‐KUB	Scanning beyond recommended anatomical boundaries: Chest CT and CTPA: Superior edge of the first rib to the costophrenic sinuses Abdominopelvic: Dome of the diaphragm to the tuber ischiadicum Chest‐ abdomin‐pelvis: Superior edge of the first rib to the tuber ischiadicum CT‐KUB: T10 vertebral level to the tuber ischiadicum Acceptable tolerance: 20 mm per direction	Retrospective analysis of multiplanar reformatted CT images: excess scan length measured as the difference between actual and standard coverage
Zanca et al. [50] 2012	Chest CT, abdominal CT and chest–abdominal CT	Scanning beyond the prescribed anatomical margins: Chest: lung apices to costodiaphragmatic sinus Abdomen: diaphragm dome to inferior pubic rami Acceptable tolerance: 20 mm per direction	Retrospective analysis of multiplanar reformatted CT images: excess scan length measured as the difference between planned coverage on the topogram and actual coverage on coronal images
Liao et al. [51] 2011	Chest, abdomen and/or pelvis CT	Any imaging beyond the protocol‐defined anatomical boundaries: Chest: Lung apices to lung base Abdomen: Diaphragm to Iliac crest Abdomen pelvis: Diaphragm to Lesser trochanters Acceptable tolerance: 20 mm per direction	Retrospective analysis of axial CT images: excess scan length measured as the difference between actual and standard coverage using predefined anatomical landmarks
Rodrigues et al. [52] 2013	CTPA	Imaging beyond lung apices and bases Acceptable tolerance: None or 10 mm per direction	Retrospective analysis of multiplanar reformatted CT images: manual measurement of scan length using predefined anatomical landmarks, comparing planning based on frontal topogram alone versus frontal + lateral topograms
Leschka et al. [53] 2010	Coronary CT angiography(CCTA)	Imaging beyond the coronary arteries (carina to cardiac apex) Acceptable tolerance: 10 mm per direction	Prospective analysis of axial CT images: manual measurement of scan length using predefined anatomical landmarks, comparing planning based on scout view versus calcium scoring images
Duerden et al. [54] 2022	CCTA	Any scan length extending beyond the coronary anatomy (from the carina to the cardiac apex) Acceptable tolerance: 10 mm per direction	Retrospective analysis of multiplanar reformatted CT images: manual measurement of scan length using predefined anatomical landmarks, comparing planning based on ultra‐low‐dose tin‐filter scout versus localiser alone
Demircioğlu et al. [55] 2024	CCTA	Scan range exceeding the optimal anatomical boundaries (from the proximal ascending aorta to the apex of the heart) Acceptable tolerance: 10 mm per direction	Deep learning‐based scan range optimisation for coronary CT angiography: VFNet neural network trained on CT localisers to predict scan start and end positions; automated range compared to radiographers' manual planning to evaluate over‐scanning
Young et al. [56] 2020	CCTA	Imaging beyond proximal and mid‐coronary arteries (carina to the greatest convexity of the right atrium) Acceptable tolerance: 10 mm per direction	Retrospective analysis of axial CT images: manual measurement of scan length using predefined anatomical landmarks, comparing full scan length versus simulated reduced scan length

Abbreviations: ALD, anatomic landmark detection; ANN, artificial neural network; CCTA, coronary CT angiography; CGAN, conditional generative adversarial network; CNN, convolutional neural network; CT, computed tomography; CTC, CT colonography; CTDI_vol_, volume CT dose index; CT‐KUB, computed tomography of the kidneys, ureters and bladder; CTPA, CT pulmonary angiography; DLP, dose length product.

### 3.3 Over‐Scanning Extent, Prevalence and Radiation Dose

Across the included studies, over‐scanning was consistently observed in all CT protocol groups, although the magnitude and frequency varied by anatomical region. Excess coverage in brain CT typically ranged from approximately 20–35 mm [19, 20, 21, 22], whereas chest CT demonstrated the greatest variability, extending from about 12 mm in optimised low‐dose examinations to more than 75 mm in routine diagnostic protocols [33, 34, 35, 36, 37, 38, 39, 40, 41, 42, 43]. Abdominal and abdominopelvic imaging generally showed excess lengths between roughly 15 and 68 mm [44, 45, 46, 47], while CT‐KUB frequently exhibited the largest extensions, often more than 50 mm and up to 75 mm in some audits [24, 25, 26, 27, 28, 29, 30, 31, 32]. Specialised examinations demonstrated comparable patterns: CTPA commonly showed excess coverage of approximately 47 mm, and CCTA ranged from about 12 to more than 80 mm [52, 53, 54, 55]. Prevalence patterns mirrored these ranges: overscanning occurred in approximately 20%–90% of brain and chest CT examinations and remained consistently high in specialised protocols [22, 37]. CTPA typically demonstrated prevalence rates between 70% and 100%, while CCTA showed similarly high values, with several studies reporting prevalence close to universal [52, 53, 54, 55]. Overscanning was almost universal in CT‐KUB and paediatric abdominopelvic imaging [32, 49]. Multi‐region examinations, such as chest–abdomen–pelvis CT, also demonstrated high prevalence, up to 90%, indicating substantial cumulative excess coverage across combined anatomical regions [48, 51].

Radiation‐dose increases attributable to over‐scanning varied markedly across protocols, reflecting differences in scan length extension and inherent protocol dose. In brain CT, excess effective dose was generally below 1 mSv [19, 20, 21, 22], whereas chest CT demonstrated the widest variation [33, 34, 35, 36, 37, 38, 39, 40, 41, 42, 43], with increases ranging from negligible values in optimised low‐dose screening examinations to more than 2 mSv in standard diagnostic protocols. Abdominal and abdominopelvic CT typically showed excess doses of approximately 0.5–2.6 mSv, with low‐dose CT colonography reporting 0.37 mSv [44, 45, 46, 47]. CT‐KUB exhibited some of the highest protocol‐specific increases, with several studies reporting additional effective doses up to 3.4 mSv [24, 25, 26, 27, 28, 29, 30, 31, 32]. Specialised examinations followed similar trends: CTPA demonstrated excess doses of around 0.7–1.2 mSv, and CCTA showed increases typically between 0.7 and 1.7 mSv [52, 53, 54, 55]. Organ‐dose findings revealed disproportionate effects on radiosensitive tissues, with substantial increases reported for the thyroid, lungs and gonads, particularly in paediatric examinations [21, 22, 47, 50]. Across all protocols, these dose escalations indicate that even modest extensions in scan range can translate into clinically meaningful increases in both effective and organ‐specific dose. Detailed findings from the 38 included studies, including extent, prevalence and associated dose estimates, are presented in Table 3.

**TABLE 3:** Extent of over‐scanning, prevalence, radiation dose and direction.

Author(s) and year	Over‐scanning extent (mm or % of scan length)	Prevalence (%)	Predominant over‐scanning boundary	Radiation dose (mSv)	Outcomes and recommendations
Zadeh et al. [19] 2025	34.4 mm	77.5%	Inferior	NR	Over‐scanning occurred exclusively in the caudal direction, increasing radiation exposure to non‐target organs: lungs +308%, thyroid +299%, oesophagus +255%, salivary glands and oral mucosa Thyroid cancer risk rose by 254% in females and 824% in children aged 5–10 years
Woo et al. [20] 2013	NR	22.2%	Inferior	NR	Over‐scanning beyond the region of interest was a frequent error, particularly after the installation of new CT machines
Botwe et al. [21] 2022	Brain: 28.6 mm Lung: 58.1 mm CTPA: 46.7 mm Abdominopelvic: 44.6 mm	70.6%	Brain & Chest: Inferior CTPA & Abdominopelvic: Superior	Brain: 0.3 Lung: 0.64 CTPA: 0.71 Abdominopelvic: 0.71	Over‐scanning significantly increases organ dose Optimisation can reduce DLP by up to 18.8% Impact of eliminating over‐scanning: DLP reduction: Brain 17.5%; Chest 18.8%; CTPA 15.5%; Abdominopelvic 9.0%. Organ dose reduction: 0.8%–79.1%
Ampofo et al. [22] 2025	Head 23.4 mm Chest 56.4 mm Abdominopelvic: 67.5 mm	Overall 87.3% Head: 88.7% Chest: 77.6% Abdominopelvic: 97.7%	Inferior	Head 0.97 Chest 0.38 Abdominopelvic: 1.52	Over‐scanning linked to increased cancer risk in children Optimisation outcomes when over‐scanning was prevented: Chest CT (Neonates): DLP reduction 27.9%; Thyroid dose reduction 57.5% Chest CT (Children): DLP reduction 26.1%; Lung dose reduction 63.6% Abdominopelvic CT (Infants): DLP reduction 26.2%; Testes dose reduction 79.98%
Badawy et al. [23] 2019	Audit: 33 mm Re‐audit (1): 23 mm Re‐audit (2): 22 mm	Audit: 58% Re‐audit (1): 33% Re‐audit (2): 27%	Inferior	Audit: 2.8 Re‐audit (1): 2.3 Re‐audit (2): 2.2	Awareness training reduced over‐scanning by 11 mm (33 → 22 mm), frequency decreased by 31%, DLP reduced by 100 mGy·cm, effective dose reduced by 0.6 mSv
Blom et al. [24] 2025	Audit: 60 mm Re‐audit: 12 mm	Audit: 80% Re‐audit: 20%	Superior	Audit: 0.59 Re‐audit: 0.27	Sagittal scout view and osseous landmarks (T10) reduced scan length by 20% and DLP by 55%
Alrosan et al. [25] 2024	NR	100%	Superior	NR	Audit revealed complete non‐compliance with best‐practice guidelines, resulting in unnecessary radiation exposure
Zafar et al. [26] 2024	NR	Audit: 83.4% Re‐audit: 37.1%	NR	NR	Re‐audit after adopting upper pole of right kidney as superior limit (instead of T10) reduced over‐scanned cases by 46.3%
Owien et al. [27] 2024	NR	68.9%	Superior	1.30	Using T10 vertebra and pubic symphysis as anatomical landmarks improved scan length accuracy compared to diaphragm‐based planning
Ghoshal et al. [28] 2021	72.12 mm	89.8%	Superior	NR	Modifying the superior landmark from T10 to T11, with the pubic symphysis as the inferior limit, improved consistency and reduced unnecessary coverage
Kasi et al. [29] 2021	53.8 mm Superior: 40–50 mm Inferior 10–20 mm	94.7%	Superior	0.41 Superior 0.5 Inferior 0.32	Phantom simulations showed that superior over‐scanning increased DLP by 2%–35%, while inferior over‐scanning raised DLP by 1%–45% Even minimal inferior extension caused proportionally greater dose escalation due to anatomical density
Netke et al. [30] 2020	Audit: 15.96% Re‐audit: 12.49%	Audit: 81% Re‐audit: 14%	Superior	Audit: 0.58	Real‐time image monitoring and manual termination at the upper pole of the highest kidney reduced over‐scanned cases to 14%, with mean excess coverage decreasing from 15.96% to 12.49%
Uldin et al. [31] 2020	Audit: 28.2% Re‐audit: 10.6%	Audit: 94.4% Re‐audit: 35.2%	Superior	NR	Re‐audit after changing the superior landmark from T10 to T11 (inferior limit: pubic symphysis) achieved a 62.4% reduction in over‐scanning without under‐scanning
Leon et al. [32] 2014	Mean: 74 mm Superior: 40.3 mm Inferior: 33.7 mm	100%	Superior	3.4	Scanning beyond defined *z*‐axis boundaries was common in CT for suspected urolithiasis; initiating CT‐KUB scans at T11 may reduce unnecessary coverage
Ebrahimian et al. [33] 2021	20 mm	63.3%	Inferior	1.3	Over‐scanning and mis‐centring were frequent across four international facilities, resulting in increased radiation dose Raising arms was associated with shorter scan lengths and lower dose
Cohen et al. [34] 2020	33 mm Superior: 5 mm Inferior: 29 mm	88%	Inferior	NR	Patient‐specific factors such as age, body size and positioning influenced scan length Automated *z*‐axis coverage tools offer potential for improving
Schwartz et al. [35] 2018	Mean: 27.2% Inferior: 7.5% Superior: 24.5%	NR	Inferior	0.29	Using coronal and sagittal scouts reduced over‐scanning Simultaneous cranial and caudal over‐scanning increased the effective dose from 0.29 mSv (with 2 cm margin) to 0.6 mSv (without a margin). Organ effective dose increases included thyroid (+0.35 mSv) and upper abdominal organs (up to +14%)
Bang et al. [36] 2013	AP protocol: 84.85 mm Lat protocol: 58.50 mm AP & Lat protocol: 63.64 mm	NR	Inferior	AP: 0.76 Lat protocol: 0.74 AP & Lat protocol: 0.77	Use of a lateral scout instead of AP alone reduced dose by 0.028 mSv; compared to combined AP and lateral views, lateral alone lowered dose by 0.038 mSv. Additional dose from lateral topogram was minimal (0.13 mSv)
Salimi et al. [37] 2021	51.4 mm Superior: 20.2 mm Inferior: 31.2 mm	99%	Inferior	2.6	Deep learning‐based automated scan range selection reduced scan length by approximately 31 mm and radiation dose by 1.3 mSv (≈17% decrease in effective dose) Over‐scanning increased thyroid absorbed dose by 67%
Huo et al. [38] 2019	58.5 mm Superior: 17 mm Inferior: 41 mm	NR	Inferior	0.72	Machine learning‐based pre‐scan boundary prediction demonstrated potential to improve scan planning and support quality control by monitoring and optimising scan length
Colevray et al. [39] 2019	74.5 mm Superior: 23.3 mm Inferior: 51.2 mm	87.5%	Inferior	NR	Deep learning‐based automated boundary detection with real‐time feedback improved scan precision and reduced over‐scanning; the model achieved 98.4% classification accuracy with strong agreement to radiologists
Demircioğlu et al. [40] 2021	29 mm	NR	Inferior	0.25	Conditional GAN‐based approach accurately automated scan range delimitation from topograms, reducing scan time, over‐scanning and radiation exposure, including a thyroid effective dose reduction of 17.7%
Kaviani et al. [41] 2022	60.3 mm Superior: 19.8 mm Inferior: 40.5 mm	NR	Inferior	NR	Deep learning models achieved 98%–100% accuracy in monitoring scan length, with no performance variation by age, gender or scanner type
Ruan et al. [42] 2022	16.2 mm	57.1%	Inferior	0.04	Automated boundary prediction using deep learning reduced over‐scanning and radiation dose, with rapid processing (~0.25 s per scan), eliminating the need for lateral scouts
Kim et al. [43] 2022	16.3 mm	24.25%	NR	0.03	Retrospective monitoring using deep learning algorithms in LDCT screening identified unnecessary scan extension and supported consistent scan planning
Campelo et al. [44] 2020	NR	13.5%	NR	NR	Lack of optimisation and protocol standardisation contributed to up to threefold differences in CTDI_vol_ between institutions; over‐scanning and repeated acquisitions were major sources of excess dose
Luu et al. [45] 2022	Abdomen: 250 mm Liver 27.8 mm	NR	Inferior	Abdomen: 2.6	Large reduction in scan range observed when automatic liver‐focused planning replaced full‐abdomen manual planning; CNN + LRS + motion modelling reduced excess radiation without compromising diagnostic quality and achieved rapid processing (~0.5 s)
Zhang et al. [46] 2015	NR	92.4%	NR	NR	A neural network‐based QA tool achieved 92.4% classification accuracy, with potential for real‐time integration into the scanner workflow
Salerno et al. [47] 2019	35.5 mm	NR	Inferior	0.37	Over‐scanning below the anal orifice increased effective dose by 6% at 2 cm and 12% at 5 cm. Male gonads were most affected, with organ equivalent dose for the testes increasing by 2–12 mSv (mean 3 mSv) and for the prostate by 4–28 mSv (mean 9 mSv). Female impact was minimal, with up to 8 cm extension increasing effective dose by ≤ 2%
Golbus et al. [48] 2024	Chest: 12.7 mm Abdomen: 14.5 mm Chest/abdomen/pelvis: 25 mm	79.80%	NR	Chest: 0.3 Abdomen: 1.7 Chest/abdomen/pelvis: 2.2	AI‐based planning using 3D landmark scan and anatomic landmark detection reduced scout dose by 3 mGy·cm, acquisition dose by up to 2.2 mSv (23.3%) and over‐scanning by 26.7 mm; planning completed in seconds without missed anatomy
Yar et al. [49] 2021	NR	Thorax CT: 89.2% Abdominopelvic: 45.6% Chest‐ abdomin‐pelvis: 42.6% CT‐KUB: 8.1% CTPA: 87%	Chest, chest‐abdominopelvic & CTPA: Inferior Abdominopelvic & CT‐KUB: Superior	Thorax: 0.37 Abdominopelvic: 0.6 Chest‐ abdomin‐pelvis: 0.9 CTPA: 1.16 CT‐KUB: 0.08	Only 2.7% of over‐scanned areas revealed clinically significant incidental findings Over‐scanning was most frequent in chest CT (89.2%) and least in urinary stone protocol CT (8.1%), adding unnecessary radiation without diagnostic benefit
Zanca et al. [50] 2012	Chest: 47 mm Superior: 18 mm Inferior: 29 mm Abdomen: 46 mm Superior: 21 mm Inferior: 25 mm	Overall: 80% Chest CT: 67% Abdomen CT: 45%	Inferior	Chest–Abdomen: 0.9 Chest: 0.6 Abdomen: 0.5 mSv	Over‐scanning contributed to organ doses within ranges associated with increased cancer risk Organ equivalent dose increases were significant (*p* < 0.001): thyroid +99% (5.1 mSv), liver +56% (2.2 mSv), testes +115% (7.6 mSv), breasts +163% (1.5 mSv) In multi‐phase protocols, excess dose was multiplied, reaching up to three times higher in triple‐phase scans
Liao et al. [51] 2011	Chest: 48 mm Abdomenopelvic: 42 mm chest‐abdominopelvic: 30 mm	99%	Inferior	0.89	Over‐scanning at the superior boundary (lung apices, diaphragm) added 26.95–38.03 mGy·cm to DLP, while inferior extension (lung bases, pelvis) added 18.85–40.89 mGy·cm, increasing exposure to radiosensitive organs including thyroid, lungs, breasts and pelvic structures
Rodrigues et al. [52] 2013	AP protocol: 39.1 mm AP & Lat protocol: 19.5 mm	AP protocol: 100% AP & Lat: 86% When allowing a 20 mm margin: AP only: 78% AP & Lat: 34%	Inferior	NR	Adding a lateral topogram to the frontal view reduced over‐scanning by 19.6 mm at the inferior boundary Associated organ dose reductions: liver 0.78 mGy, stomach 0.98 mGy, thyroid 0.83 mGy Effective dose remained similar (frontal only: 6.0 ± 2.4 mSv; frontal + lateral: 6.1 ± 1.8 mSv) Additional dose from the lateral topogram was minimal (0.13 mSv)
Leschka et al. [53] 2010	21 mm	99.2%	Superior	1.7	Using calcium scoring images to define scan range reduced over‐scanning by approximately 21 mm (≈15%) and radiation dose by 1.7 mSv (≈16%), while maintaining complete coronary artery visualisation
Duerden et al. [54] 2022	12 mm	NR	Inferior	NR	Inclusion of an ultra‐low dose planning scan shortened total scan length (124 ± 13 mm vs. 117 ± 13 mm, *p* = 0.007), reduced excess coverage (12 ± 9 mm vs. 5 ± 4 mm, *p* = 0.001) and lowered caudal excess (11 ± 8 mm vs. 6 ± 4 mm), with no increase in radiation dose (2.1 mSv vs. 2.2 mSv)
Demircioğlu et al. [55] 2024	45.6 mm	NR	NR	0.9	Automated scan range planning reduced scan length by 15–20 mm and radiation dose from 0.9 mSv (12.6%) to 0.8 mSv (10.0%), while maintaining complete coronary artery imaging in > 99% of cases
Young et al. [56] 2020	86 mm	100%	Inferior	0.67	Reduced scan length protocol (1 cm below carina to the greatest convexity of right atrium) decreased over‐scanning by about 86 mm (≈60%) and radiation dose by 0.67 mSv (≈60%), without compromising diagnostic adequacy

Abbreviations: AI, artificial intelligence; AP, anteroposterior; CCTA, coronary CT angiography; CNN, convolutional neural network; CT‐KUB, computed tomography of the kidneys; CTPA, CT pulmonary angiography; DLP, dose‐length product; GAN, generative adversarial network; Lat, lateral; LDCT, low‐dose computed tomography; LRS, liver range search; NR, not reported; QA, quality assurance.

Paediatric examinations consistently showed greater proportional dose impact for a comparable extent of excess coverage than adult examinations. Across protocols, paediatric head CT generally demonstrated excess coverage between 23 and 34 mm, with prevalence 75%–90%, compared with 20–30 mm and about 70% prevalence in adult audits [19, 22]. In paediatric chest CT, excess lengths 55–60 mm were common, with prevalence about 75%; adult chest CT showed wider variability 12–85 mm, with prevalence 25%–99% [21, 49]. For abdominopelvic imaging, paediatric excess coverage often reached 65–70 mm, with very high prevalence 95% or higher; adult abdominopelvic or combined chest–abdomen examinations typically showed 40–50 mm, with prevalence 45%–99% [50, 51]. Despite heterogeneity in definitions and dosimetric methods, the evidence consistently shows that children experience higher organ‐dose increases for similar excess lengths, reflecting smaller body dimensions and higher radiosensitivity [19, 22]. These findings support adopting stricter tolerance margins and paediatric specific planning rules for scan range planning.

Where age bands were reported, over‐scanning and dose impacts varied across paediatric subgroups. In paediatric head CT, caudal excess coverage was consistently observed across early childhood groups, with typical extents of about 34 mm in 0–5 years, 36 mm in 5–10 years and 32 mm in 10–15 years. These excess lengths were associated with large organ‐dose increases, including lungs (+308%), thyroid (+299%) and oesophagus (+255%), and the largest proportional rise in thyroid lifetime attributable risk was reported in children aged 5–10 years (+824%) [19]. In paediatric chest CT, redundant coverage occurred in about 77% of examinations; reducing the excess lowered DLP by 27.8% in neonates and 26% in middle childhood, with organ‐dose reductions of 57% for thyroid, 56% for the heart wall, 63% for lungs and 73% for breasts [22]. In paediatric abdominopelvic CT, overscanning was near universal (97%), with the greatest excess lengths in infants and optimisation resulted in large gonadal‐dose reductions, including a reduction in testes dose of about 80% [22]. Collectively, these findings highlight the importance of age‐specific boundary definition, conservative paediatric tolerance margins and proactive optimisation practices such as protocol standardisation, staff training and prospective boundary verification.

### 3.4 Over‐Scanning Directional Trend (Superior vs. Inferior)

Although over‐scanning was observed at both the superior and inferior boundaries, the predominant direction varied by protocol (Table 3). Over‐scanning most frequently involved the inferior boundary in brain, neck, chest, abdominal and multi‐region examinations [20, 41]. This pattern likely reflects challenges in delineating inferior anatomical limits on scout images and a conservative approach to ensure complete coverage [23, 51]. Superior over‐scanning was reported most consistently in CT‐KUB and was also described in some adult abdominopelvic and CTPA examinations [24, 49]. In paediatric imaging, similar trends were evident: inferior over‐scanning predominated in paediatric brain and chest CT, whereas superior excess coverage was more frequently observed in paediatric abdominopelvic examinations [19, 22]. Overall, the direction of over‐scanning appears protocol‐dependent and is influenced by anatomical landmark visibility and cautious boundary selection at the superior and inferior boundaries.

### 3.5 Contributing Factors and Mitigation Strategies

Across the included studies, multiple factors contributed to over‐scanning in CT. Protocol‐level issues were most frequent (36.7%), followed by operator‐level (33.7%), system‐level (20.1%) and patient‐level (9.5%). Within the operator‐level, radiographer conservatism or caution in scan planning was the dominant contributor (45.6%), followed by variability in anatomical boundary estimation (26.3%), less experience (14.0%), lack of dose awareness (7.0%) and non‐compliance with scan length guidelines (7.0%) [25, 36]. Protocol‐level factors included: absence of standardised anatomical landmarks (33.9%), scout image limitations (lacking cross‐sectional anatomical detail) (25.8%), lack of sagittal scout views (16.1%), use of soft‐tissue landmarks (12.9%) and absence of tailored protocols (11.3%) [29, 31]. System‐level contributors were primarily linked to the absence of automated scan range planning (41.2%), lack of audit, training, or feedback (35.3%) and work environment pressures such as high workload and teaching settings (23.5%) [26, 42]. Patient‐level factors included anatomical and pathological variation (56.3%), which often related to body mass index (BMI), body habitus and breathing patterns and uncooperative or restless children (43.8%) [34, 37]. Overall, radiographer conservatism or caution in scan planning was the most frequently reported factor across all categories.

**FIGURE 2:**
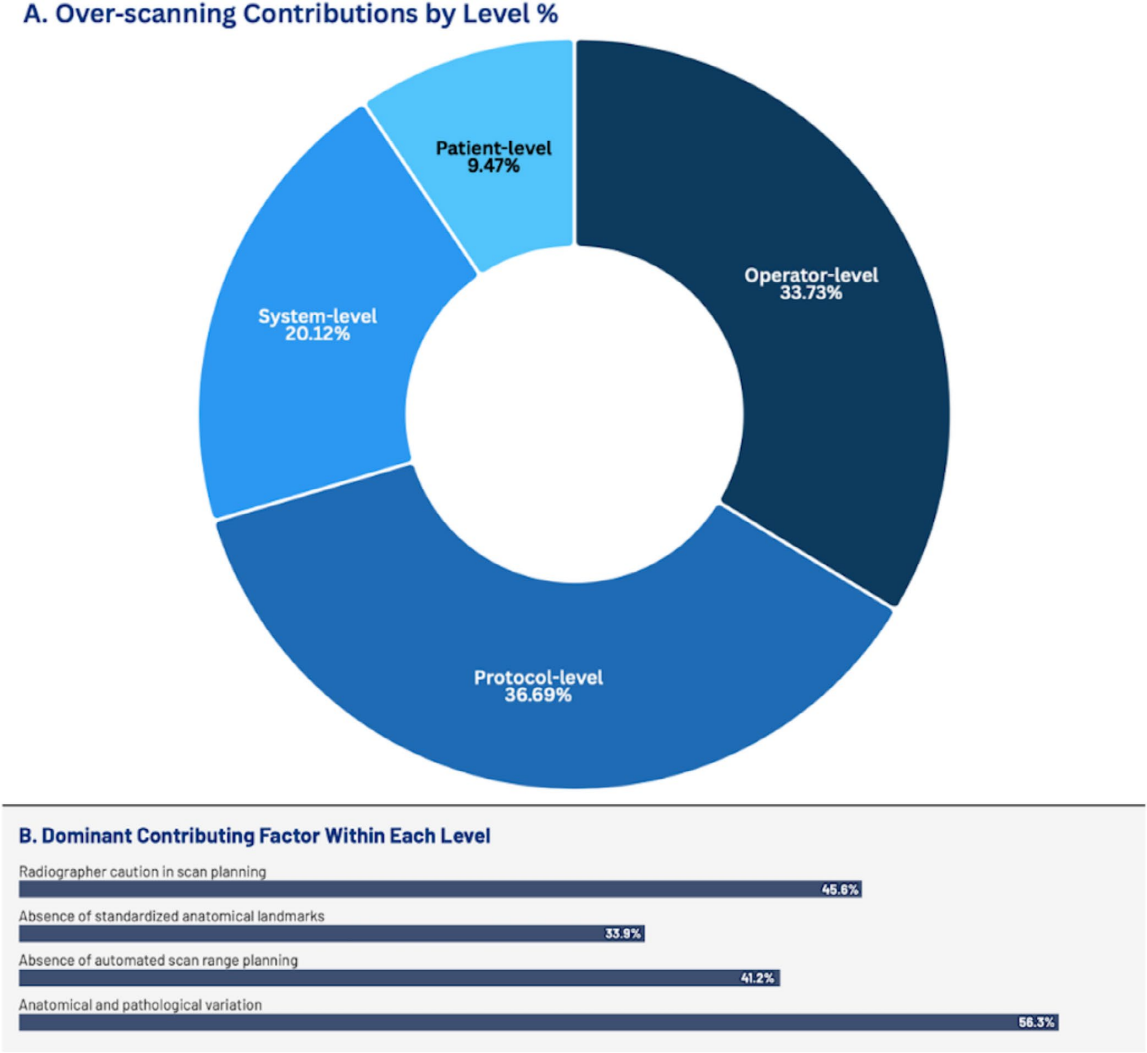
(A) Over‐scanning contributions by level (operator, protocol, system, patient). (B) Dominant contributing factor within each level.

A range of mitigation strategies was implemented and recommended to address these contributing factors at multiple levels. Operator‐level issues, such as radiographer caution and variability in boundary estimation, were commonly addressed through education, competency‐based training and personalised feedback programs [29, 30, 31]. Protocol‐level factors, including absence of standardised landmarks and scout image limitations, were mitigated by implementing clearly defined osseous landmarks, incorporating lateral scout views and tailoring protocols for paediatric patients [52, 53]. System‐level contributors, such as lack of automated planning and audit processes, were linked to strategies like integrating AI‐assisted planning tools, dose monitoring software and structured feedback systems [40, 43]. Patient‐level challenges, including anatomical variation and uncooperative children, were managed through patient‐specific positioning adjustments, clear breathing instructions and sedation protocols where appropriate [19, 22]. Figure 2 summarises contributions by level and the dominant factor within each level; Table 4 lists the most frequent factors and corresponding mitigation strategies.

**TABLE 4:** Contributing factors to over‐scanning in CT imaging and recommended mitigation strategies.

Category	Common contributing factor	Common mitigation strategies
Operator‐level	Radiographer's caution in scan planning Variability in anatomical boundary estimation Technologist inexperience and lack of training Limited radiographer awareness of dose implications Non‐compliance with scan length guidelines	Radiographer education and re‐education Structured competency‐based training programs Radiation safety awareness and dose monitoring Regular audits and feedback programs Assign complex scans to experienced staff Personalised technologist feedback programs
Protocol‐level	Absence of standardised anatomical landmarks Scout image limitations (lack of cross‐sectional anatomical detail) Lack of sagittal scout view Use of soft tissue landmarks Lack of tailored protocols (e.g., paediatric)	Protocol standardisation and supervision Use of clearly defined osseous landmarks (e.g., vertebral levels) Incorporation of lateral scout view Paediatric‐specific protocols and clinical indication‐based planning Consistent application of anatomical landmarking Protocol‐specific adjustments for complex or staging exams
System‐level	Absence of automated scan range planning Absence of audit, training, feedback, or dose monitoring Work environment factors (teaching hospital setting, high radiographer turnover, heavy workload, time pressure, on‐call duties and day scanning demands)	Integration of AI‐assisted scan range planning tools into routine workflow Regular audits and structured feedback systems Dose monitoring and tracking software Workflow optimisation and staffing adjustments
Patient‐level	Anatomical and pathological variation (including BMI, body habitus and breathing patterns) Uncooperative or restless children	Patient‐specific planning and positioning adjustments Consideration of patient‐specific factors (e.g., BMI) Clear breathing instructions Sedation protocols for paediatric patients

## 4 Discussion

This scoping review of 38 studies confirms that extending scan coverage beyond intended anatomical boundaries offers negligible diagnostic benefit. Incidental findings were rare, with clinically significant findings reported in only about 2%–3% of over‐scanned regions; when present, they were mainly small pulmonary nodules or incidental abdominal lesions and rarely altered patient management [49, 50]. These findings highlight that additional coverage does not improve diagnostic yield but increases radiation exposure, particularly in paediatric patients where radiosensitive organs heighten long‐term cancer risk [19]. Current CT dose optimisation strategies have largely focused on technical parameters such as tube current modulation and iterative reconstruction; however, this review identifies scan range selection as a critical yet under‐addressed contributor to unnecessary radiation exposure.

Evidence from the included studies reveals substantial heterogeneity in the extent and prevalence of over‐scanning among different CT protocols. Excessive coverage is not confined to complex or multi‐region examinations; it is consistently observed in routine protocols such as brain, chest, abdominal and abdominopelvic CT as well as in targeted studies like CT‐KUB, CTPA and cardiac CT [48, 49, 50, 51]. Reported over‐scanning ranged from approximately 12 mm to about 90 mm, with prevalence ranging from 13% to 100% across protocols. This recurring pattern of substantial excess coverage indicates that current planning practices often prioritise avoidance of under‐scanning over adherence to anatomical boundaries. In some clinical contexts, particularly acute presentations such as suspected renal colic, CT‐KUB coverage may be deliberately extended to include the full abdomen and pelvis to avoid missing alternative diagnoses. While clinically justified, this approach still increases radiation exposure and highlights the need to balance diagnostic caution with optimised scan range selection. Such variability underscores the absence of universally applied tolerance margins and highlights the need for harmonised standards that balance diagnostic adequacy with radiation safety. The mean over‐scanning prevalence across studies was approximately 75%, reflecting a systemic issue rather than isolated error and signalling that current CT practice tolerates substantial excess coverage despite decades of dose‐optimisation initiatives.

Across the included studies, additional coverage was associated with effective dose increases ranging from fractions of a millisievert in low‐dose protocols to more than 3 mSv in some abdominal and CT‐KUB examinations. These values, although seemingly modest in isolation, represent a considerable cumulative burden, particularly for patients undergoing repeated imaging or paediatric cohorts. In multiphase CT acquisitions, even small extensions in scan range may be propagated across sequential phases, thereby amplifying cumulative radiation exposure. Organ‐specific dose escalations have been reported to increase several‐fold, notably affecting radiosensitive organs such as the thyroid, lungs and gonads, and amplifying lifetime cancer risk [19, 22]. Over‐scanning at cranial and caudal boundaries further compounds this effect, with inferior extensions exerting greater dose impact due to anatomical density and proximity of critical organs [11]. Evidence suggests that strict adherence to anatomical boundaries can reduce organ doses by up to 79%, highlighting the value of implementing real‐time planning tools and audit‐driven protocols to mitigate unnecessary radiation exposure [21, 47].

Contributing factors to over‐scanning span protocol, operator, system and patient domains. Protocol‐level issues, such as the absence of standardised anatomical landmarks and reliance on soft tissue references, were the most frequent drivers, often compounded by limited visibility on scout images [26, 31]. Operator‐level factors included radiographer caution, experience level, variability in boundary estimation and inconsistent adherence to paediatric‐specific protocols, particularly in high‐pressure settings [23, 27]. System‐level contributors, such as lack of automated planning tools, audit cycles and structured feedback, were amplified by workflow pressures and high staff turnover [34, 37]. Patient‐related factors, including anatomical variability, motion artefacts and positioning errors, further increased scan range uncertainty [28, 53]. Collectively, these findings underscore that over‐scanning reflects an interplay of technical, human and systemic factors, necessitating multi‐level interventions to achieve sustainable dose optimisation.

Directional patterns of over‐scanning varied across protocols but demonstrated consistent tendencies linked to anatomical visibility and radiographer caution [21, 47]. Inferior extensions predominated in brain, chest and multi‐region examinations, largely due to poor visualisation of caudal landmarks on scout views and conservative planning to avoid under‐coverage [34, 56]. Conversely, superior over‐scanning was more frequent in CT‐KUB and certain abdominopelvic protocols, reflecting variability in cranial landmarks such as the diaphragm [24, 26]. These trends underscore the influence of anatomical complexity and landmark visibility on scan planning practices and reinforce the need for standardised boundary definitions to reduce variability [27, 30].

The concept of acceptable tolerance margins for scan range extension remains inconsistent across protocols [34, 56]. Adult studies often apply margins of approximately 20 mm when defining acceptable limits to accommodate respiratory motion and operator variability [35, 50]. In contrast, paediatric protocols recommend stricter limits of 10 mm or less [19, 22]. Applying these margins reduces the likelihood of classifying scans as over‐scanned. However, removing them has been associated with substantial increases in organ dose [30]. Despite proposed limits, adherence remains poor and no universally accepted standard exists, underscoring the need for clear, evidence‐based guidelines to balance diagnostic adequacy with radiation safety.

Mitigating over‐scanning requires a multi‐level approach. Evidence‐based measures include using standardised anatomical landmarks, adapted for patient‐specific factors such as anatomical variation and paediatric protocols, along with additional scout views to improve boundary identification and minimise unnecessary coverage [20, 21, 22]. Protocol‐level interventions, such as standardised osseous landmarks and lateral scout views, have demonstrated substantial reductions in over‐scanning [20, 21, 22]. Operator‐focused strategies, including structured education, competency‐based training and regular audit‐feedback cycles, further enhance adherence to optimised protocols [23, 31]. System‐level solutions, notably the integration of AI‐assisted planning tools and real‐time feedback mechanisms, offer promising avenues for automation and consistency [39, 43]. Recent evidence shows that AI‐based approaches can reduce scan length by 15–71 mm and radiation dose by 5%–47%, while improving anatomical coverage accuracy and minimising inter‐operator variability [15]. These tools provide dual functionality: real‐time decision support during scan planning and retrospective audit capabilities to quantify excess coverage and inform protocol optimisation [14]. Successful implementation, however, depends on robust external validation and radiographer oversight to ensure safe and context‐appropriate application [45, 48]. Patient‐centred measures, including proper positioning and breathing instructions, complement these efforts. Collectively, these strategies underscore the importance of combining technological innovation with standardised practice and continuous professional development to achieve sustainable dose optimisation.

This review highlights that over‐scanning in CT imaging is a pervasive and largely avoidable contributor to unnecessary radiation exposure. Its negligible diagnostic benefit, coupled with significant dose implications particularly for radiosensitive organs and paediatric populations, underscores the urgency of implementing standardised scan range protocols. Clinical practice should prioritise evidence‐based measures, including clear anatomical landmarks, optimised scout imaging and structured audit‐feedback systems supported by continuous radiographer education. These findings also have practical implications for daily workflow, emphasising the need for consistent training and integration of planning tools to help radiographers reduce unnecessary coverage, improve consistency and support safer decision‐making. Beyond workflow considerations, over‐scanning may increase data storage and archiving demands within PACS systems, representing an additional operational burden that also warrants consideration. Future research should focus on developing universally accepted tolerance margins, validating AI‐assisted planning tools across diverse clinical settings and integrating real‐time feedback mechanisms into routine workflows. These efforts are essential to ensure sustainable radiation dose optimisation, enhance patient safety and promote consistency in CT imaging worldwide.

### 4.1 Limitations

This review provides a comprehensive synthesis of over‐scanning in CT imaging across multiple protocols, integrating evidence on prevalence, dose implications, contributing factors and mitigation strategies. The use of the PRISMA‐ScR framework and a broad search strategy across four major databases enhances methodological rigour and transparency. Inclusion of emerging AI‐based approaches offers forward‐looking insights into automation and dose optimisation. However, most studies were retrospective, limiting causal inference. Considerable heterogeneity in over‐scanning definitions, anatomical boundaries and tolerance margins complicates direct comparisons; accordingly, results were synthesised using study‐level definitions and tolerances as reported and findings should be interpreted in light of this variability. Dosimetric data were often incomplete, with some studies relying on estimated rather than measured effective doses; therefore, reported dose values should be interpreted as indicative trends rather than precise measurements. Certain CT protocols were underrepresented, and the included studies employed both manual and AI‐based methods for scan range assessment, introducing methodological variability that may influence interpretation. These limitations should be considered when generalising findings and highlight the need for prospective studies and standardised definitions in future research.

## 5 Conclusion

Over‐scanning in CT imaging is a common and avoidable contributor to unnecessary radiation exposure. It is driven by technical factors, operator decisions, patient anatomy and system‐level factors. Its impact is particularly concerning for radiosensitive organs and paediatric populations. Mitigation strategies such as standardised anatomical landmarks, optimised scout imaging, radiographer education, clinical audits and AI‐assisted planning tools offer effective solutions to reduce unnecessary coverage and enhance patient safety. Implementing these measures consistently across institutions is essential to achieve sustainable radiation dose optimisation and promote best practice in CT imaging.

## Author Contributions

**Mo'men Bani‐Ahmad:** conceptualization, methodology, data curation, validation, writing – original draft. **Aoife O Sullivan:** methodology, software, data curation. **Yasser H. Hadi:** methodology, validation, software, data curation. **Laura McLaughlin:** validation, writing – review and editing, supervision. **Andrew England:** validation, writing – review and editing, supervision. **Mark McEntee:** validation, writing – review and editing, supervision, project administration. All authors read and approved the final manuscript.

## Funding

The authors have nothing to report.

## Ethics Statement

This study is a scoping review of previously published literature and did not involve human participants or identifiable patient data. Therefore, ethical approval was not required.

## Conflicts of Interest

The authors declare no conflicts of interest.

## Supporting information


**Data S1:** jmrs70104‐sup‐0001‐Supinfo.docx.

## Data Availability

The data that supports the findings of this study are available in the Supporting Information of this article.
